# Systematic Profiling of ssODN Determinants Establishes Optimized LbCas12a-Mediated HDR for Precise Genome Editing in *Flammulina filiformis*

**DOI:** 10.3390/jof12090705

**Published:** 2026-09-21

**Authors:** Dinghong Jia, Xun Liu, Di Wang, Xiaolan He, Xinmin Liang, Weihong Peng

**Affiliations:** Sichuan Institute of Edible Fungi, Sichuan Academy of Agricultural Sciences, Chengdu 610066, China; liuxun0712@163.com (X.L.); wang.di19881213@163.com (D.W.); xiaolanhe1121@aliyun.com (X.H.); liangxinmin_lxm@163.com (X.L.)

**Keywords:** *Flammulina filiformis*, CRISPR-LbCas12a, HDR, ssODN, PAM mutation

## Abstract

Although gene editing technologies have advanced considerably, research on gene editing in *Flammulina filiformis* remains in its infancy, and a systematic evaluation of how ssODN donor properties affect HDR efficiency in this species is still lacking. To enable precise genome editing in the edible mushroom *F. filiformis*, we established an LbCas12a ribonucleoprotein (RNP)-mediated homology-directed repair (HDR) platform in protoplasts and systematically evaluated key ssODN donor design parameters: PAM-site mutation, homology arm distance, RAD51-preferred sequences (RPSs), truncated Cas target sequences (tCTSs), the RS-1 enhancer, and thermodynamic stability (ΔG). A PAM-disrupting mutation greatly increased HDR efficiency, up to 58.5%. Extending the left homology arm 174 bp upstream of the break still yielded 20.2% HDR, expanding the tolerable distance. RPSs showed context-dependent effects: enhancing HDR up to 287.8% in some designs but reducing it by 61.9% in another. Both tCTS and RS-1 consistently suppressed HDR. No strong linear correlation between ΔG and HDR efficiency was observed. Collectively, from experiments conducted at a single genomic locus, our results not only establish a versatile precision-editing platform for *F. filiformis* through optimized ssODN design, but also offer tentative and transferable insights into the HDR-mediated genome editing of other heterokaryotic basidiomycetes facing similar genetic operation challenges.

## 1. Introduction

*Flammulina filiformis*, widely known as enokitake, is an industrially cultivated basidiomycete of high dietary significance. It is prized for its abundant proteins, amino acids and polysaccharides as well as diverse bioactive characteristics [1,2]. As one of the most widely consumed edible mushrooms globally, *F. filiformis* also serves as a model organism for fundamental research in edible fungi [3,4,5]. However, the genetic improvement and functional genomics of this species are severely constrained by its cellular biology: *F. filiformis* typically exists in a heterokaryotic dikaryotic state that differs fundamentally from the cellular organization of plants and animals, often rendering established genome-editing workflows non-transferable.

In parallel, genome-editing technologies have undergone transformative advances. Since the demonstration of sequence-specific gene editing with zinc finger nucleases (ZFNs) [6], the toolbox has rapidly expanded through TALENs [7] and the revolutionary CRISPR-Cas9 system [8]. More recently, base editing [9,10] and prime editing [11] have been developed to overcome the off-target effects and large protein size of Cas9, while RNA-targeting editors have further broadened the scope of precise nucleic acid manipulation [12,13,14]. These emerging technologies have delivered remarkable breakthroughs in human disease therapy and crop germplasm innovation, as exemplified by the successful treatment of β-thalassemia and sickle cell disease [15], the correction of infantile CPS1 deficiency [16], and the creation of fragrant, γ-aminobutyric acid (GABA)-enriched rice [17].

Despite this rapid progress, the translation of advanced genome-editing tools to *F. filiformis* has lagged conspicuously. To date, only a handful of gene-editing studies have been reported in this species, and the established CRISPR-Cas9 systems have been used almost exclusively to generate simple *pyrG* deletions or small insertions/deletions (indels). Notably, the CRISPR-Cas12a (Cpf1) system, which generates staggered DNA ends that are potentially more favorable for homology-directed repair (HDR), remains entirely unexplored in this fungus. Moreover, for precise genome modification via HDR, the influence of ssODN donor design features, such as PAM-site disruption, homology arm-to-cleavage site distance, RAD51-preferred sequences (RPSs), truncated Cas target sequences (tCTSs), and the RS-1 enhancer, has not been systematically characterized in *F. filiformis*. These parameters critically determine editing efficiency in mammalian and plant systems, yet their effects in dikaryotic basidiomycetes are unknown, constituting a major knowledge gap that hampers the adoption of precision editing in edible mushrooms.

To address this gap, we employed *F. filiformis* protoplasts to establish an LbCas12a ribonucleoprotein (RNP)-mediated HDR platform and comprehensively evaluated how these ssODN properties affect HDR efficiency. Through this work, we aimed to establish a rational framework for ssODN donor design in *F. filiformis*, providing a foundation for precise genome editing in this economically important edible fungus and delivering broadly applicable insights for emerging editing technologies in other dikaryotic fungi.

## 2. Materials and Methods

### 2.1. Protoplast Preparation and Transformation

For gene editing experiments, the wild-type strain used was the dikaryon strain 22Y of *F. filiformis*, which is deposited at the Sichuan Institute of Edible Fungi, China. Mycelia were prepared by inoculating the strain into a 300 mL Erlenmeyer flask containing 100 mL of YM medium (2% glucose, 0.5% yeast extract, and 0.06% KH_2_PO_4_), followed by incubation at 22 °C with shaking at 180 rpm for 5 days. Subsequently, the mycelia were resuspended using a vortex mixer, and 1 mL of the mycelial suspension was transferred into a 500 mL flask containing 500 mL of fresh YM medium and further incubated at 22 °C and 120 rpm for an additional 5 days.

Mycelial pellets were harvested by filtration through four layers of lens paper, washed twice with 0.6 M mannitol, and then subjected to enzymatic digestion using 2% (w/v) lywallzyme (Guangdong Institute of Microbiology, Guangzhou, China). Digestion was carried out at 30 °C with gentle shaking at 60 rpm for approximately 3 h. The resulting protoplasts were collected by filtration through six layers of lens paper.

For protoplast transformation, ssODNs, Triton X-100, and RS-1 were added to the transformation mixture at final concentrations of 0.435 pmol/μL, 0.01% (w/v), and 12 μmol/L, respectively, according to the experimental design. All other reagents and transformation procedures were performed as previously described [18].

### 2.2. Determination of Target Gene and Editing Site

**Figure 1:**
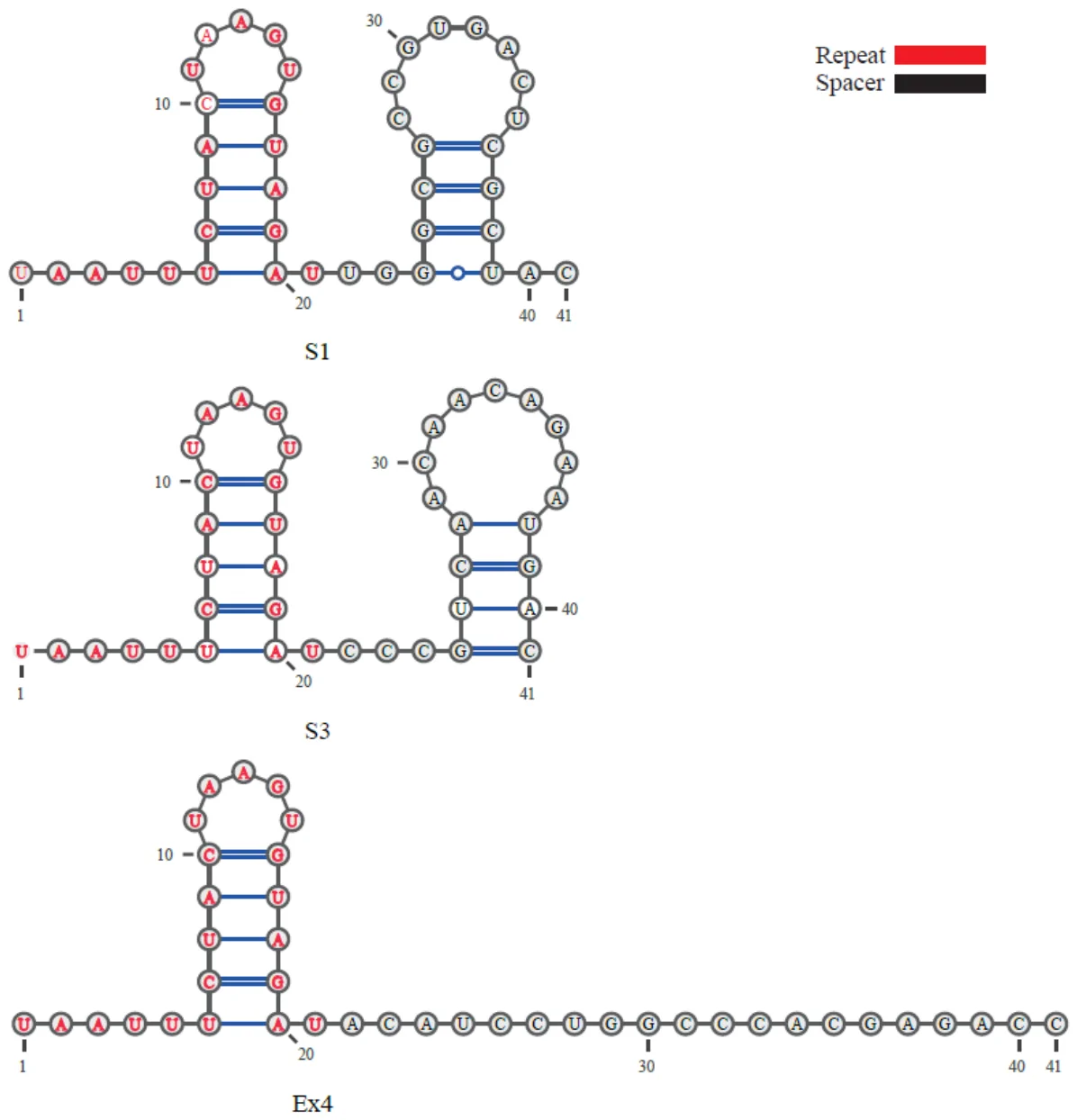
Characterization and validation of the three crRNAs. Secondary structures of crRNAs (S1, S3, and Ex4) were predicted using the RNAeval web server.

Building on prior evidence linking pigmentation in *F. filiformis* to melanin biosynthesis and tyrosinase activity [19], we cloned members of the tyrosinase gene family and identified EVM0009893 as a tyrosinase-encoding gene (*Tyr*) showing a highly significant association with coloration phenotypes. Given its significant association with coloration, EVM0009893 was selected as a target for CRISPR-mediated genome editing to establish the experimental framework and facilitate the functional validation of color-related genes. Target sites and corresponding guide RNAs were designed using CRISPR-offinder (version 1.2) (see Figure 1 for sequences) [20], and all crRNAs were synthesized by GenScript Co., Ltd. (Nanjing, China), with phosphorothioate (PS) linkages at the three terminal bases of both the 5′ and 3′ ends.

### 2.3. Screening of Lbcas12a–crRNA Variants by In Vitro Cleavage Assay

RNP complexes were assembled in an 11 µL reaction mixture containing 3.8 µL of crRNA (100 µM), 1.1 µL of 10× LbCas12a Reaction Buffer, 1.2 µL of LbCas12a nuclease (27.4 µM; GenScript, Piscataway, NJ, USA), and 4.9 µL of nuclease-free water. The mixture was incubated at room temperature for 20 min to allow for RNP formation. Subsequently, the cleavage reaction was initiated by adding 4 µL of PCR-amplified DNA fragment (~100 ng), 0.9 µL of 10× LbCas12a Reaction Buffer, and 4.1 µL of nuclease-free water to the pre-assembled RNP mixture. Following incubation at 37 °C for 1.5 h, the reaction products were analyzed by 1.5% agarose gel electrophoresis to select the most efficient LbCas12a–crRNA.

### 2.4. ssODN Design

To verify the presence and expression of the putative RAD51 gene in *F. filiformis*, quantitative PCR (qPCR) was performed. Expression levels in strain 22Y were normalized to two reference genes, DNA-directed RNA polymerase subunit 2 *(Rpb2*) and cytochrome b (*CytB*), and the primer sequences are listed in Table 1. Given that RAD51 is present and expressed in *F. filiformis*, we incorporated the RAD51-preferred sequence (RPS; 5′-AACTCCCC-3′) at the 5′ ends of the ssODN donors, as previously reported [21]. We sought to test whether this RPS motif, which performs favorably in human-derived cells, could improve ssODN-mediated HDR in our *F. filiformis* LbCas12a editing system.

**Figure 2:**
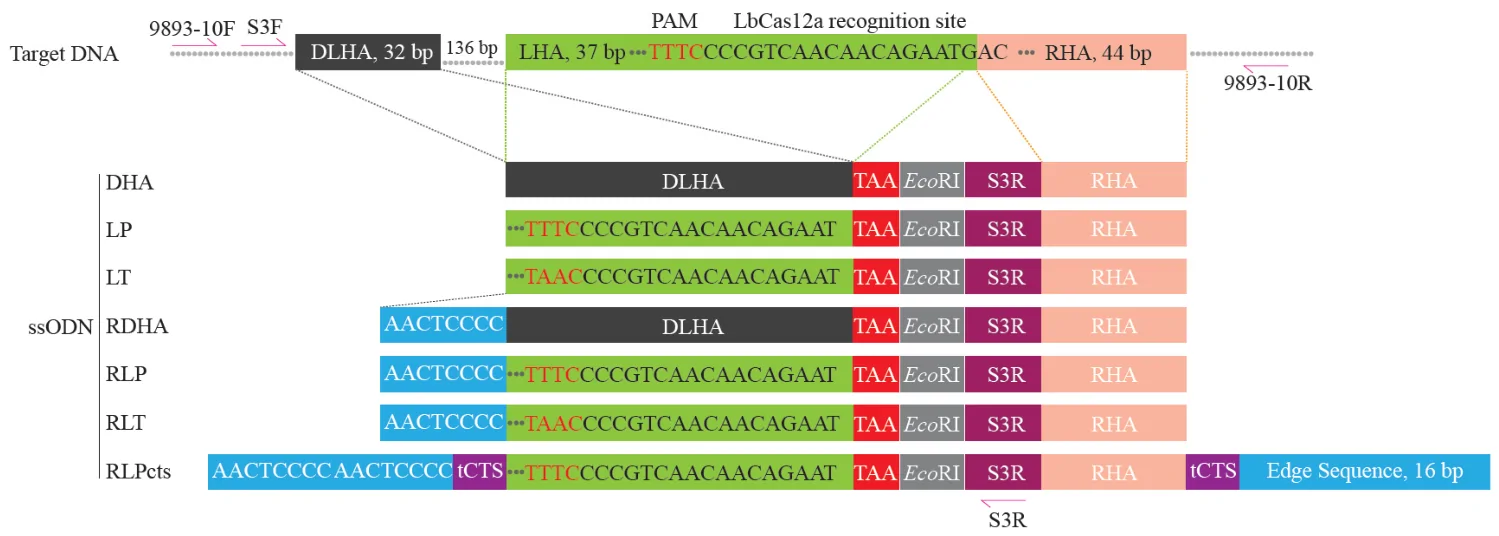
Schematic design of ssODN donor templates for HDR-mediated gene editing.

To terminate translation of the target gene and facilitate mutant screening, we inserted an engineered sequence between the left and right homologous arms. This insert comprised a stop codon, an *Eco*RI restriction site, and the reverse complement of the downstream primer S3R (manually designed), which forms a primer pair with S3F for positive screening. To evaluate the effect of tCTS sequences on recombination rates, we also introduced them at both ends of one ssODN strand. The target gene sequence and the final ssODN design are illustrated in Figure 2, with detailed nucleotide information provided in Table 1. All ssODNs were synthesized by GenScript (Nanjing) Co., Ltd. (Nanjing, China), and feature PS linkages at the two terminal bases of both the 5′ and 3′ ends. Their secondary structures and free energies were calculated using seqfold-0.10.2 [22].

### 2.5. Genomic PCR and Sequencing for Mutation Identification

To validate the targeted mutations, genomic DNA was extracted from all transformants and subjected to PCR amplification using the primer pair S3F and S3R. The resulting amplicons were purified, TA-cloned, and analyzed by Sanger sequencing to characterize variations at the edited locus. The sequences of all primers used in this study are provided in Table 1.

In this study, an “edited colony” is defined as a primary dikaryotic transformant colony harbouring at least one nucleus with the expected knock-in (KI) donor-derived sequence. For each PCR-positive transformant, six independent TA clones were Sanger-sequenced to identify mixed allelic genotypes. The reported HDR efficiencies represent the fraction of transformant colonies that contain at least one edited nuclear genotype.

Editing events are scored based on successful integration of the diagnostic inserted cassette originating from ssODN donors. This cassette harbours a stop codon, an *Eco*RI restriction site, and the reverse-complement of the manually designed downstream primer S3R. Consequently, all detected edited transformants necessarily carry the reverse-complement of S3R and correspond to bona fide KI events.

### 2.6. Statistical Analysis

For editing-efficiency comparisons, raw counts of edited and total colonies for each independent biological transformation replicate generated were determined. Editing-efficiency percentages were computed per replicate; biological replicates were kept separate and colony counts were not pooled across replicates. Overall multi-group differences were assessed by one-way ANOVA. Exact *p*-values for pairwise tests are provided in Supplementary Table S1. Error bars in bar plots denote the standard error of the mean (SEM) from three biological replicates.

For the ssODN thermodynamic stability (ΔG) versus HDR-efficiency analysis, Pearson and Spearman correlation analyses, linear regression with 95% prediction interval, Shapiro–Wilk residual normality test and subgroup Welch’s *t*-test were conducted in Python 3.9 using numpy, pandas, scipy.stats, and matplotlib.

## 3. Results

### 3.1. LbCas12a–crRNA Screening

**Figure 3:**
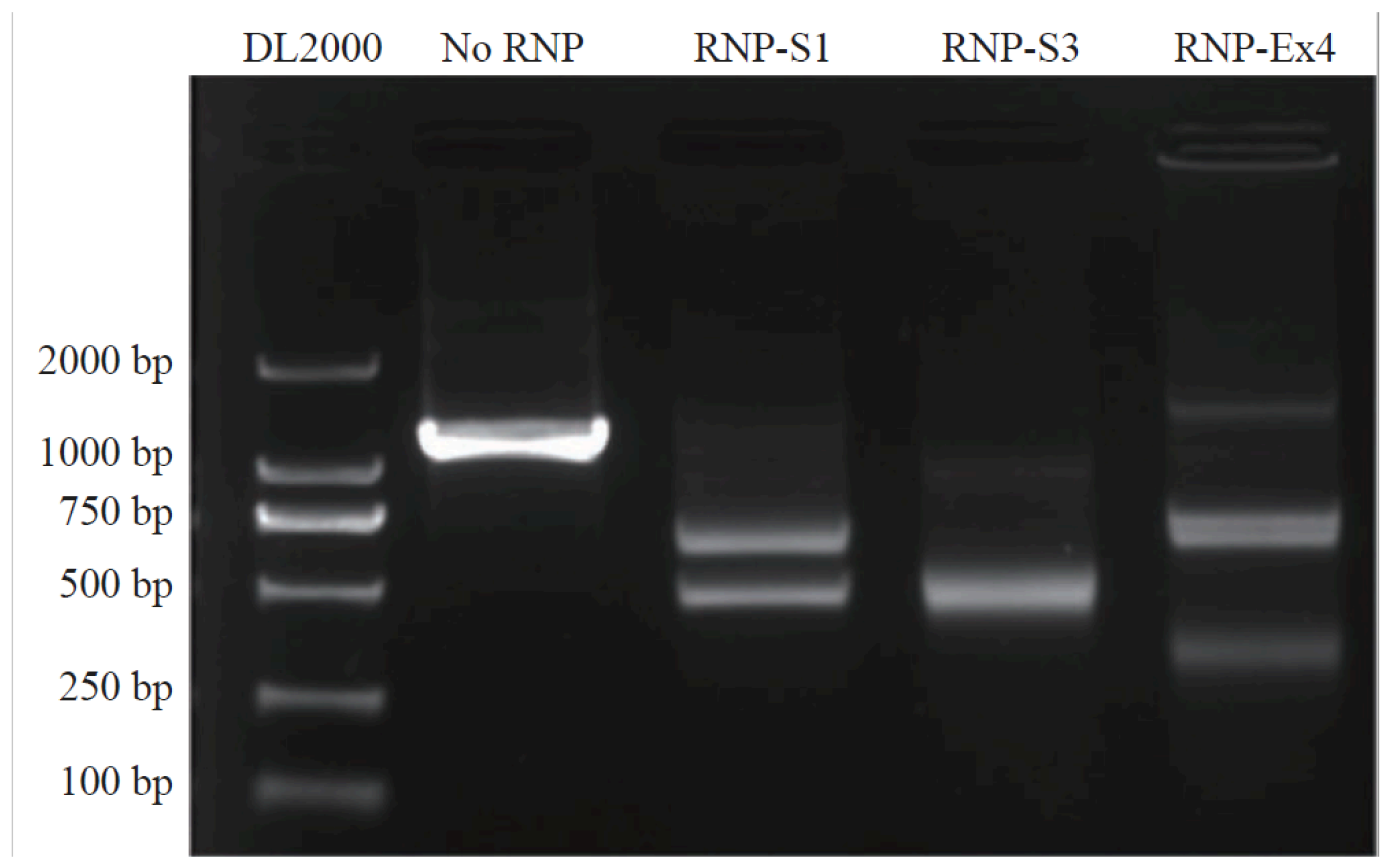
In vitro cleavage of PCR products by LbCas12a RNPs. The PCR products were incubated with the indicated pre-assembled LbCas12a RNPs at 37 °C for 1.5 h, and resolved on a 1.5% agarose gel. DL2000, DL2000 DNA marker; No RNP, substrate-only control (no LbCas12a-crRNA RNP); RNP-S1, cleavage products generated by the LbCas12a–crRNA-S1 RNP; RNP-S3, cleavage products generated by the LbCas12a–crRNA-S3 RNP; RNP-Ex4, cleavage products generated by the LbCas12a–crRNA-Ex4 RNP.

To select potential target genes, we performed an integrated association analysis of genetic variants and colorimetric phenotypes using TASSEL. This analysis revealed that the tyrosinase gene EVM0009893 contained polymorphisms significantly associated with the traits of interest (*p* < 10^−6^; Figure S1). Based on this statistical evidence, EVM0009893 was selected as the target for optimizing ssODN-mediated editing parameters. Three crRNAs were designed with CRISPR-offinder (v1.2), aiming to maintain the spacer GC content between 50% and 70% while minimizing off-target potential. To exclude candidates prone to misfolding, we predicted secondary structures using the RNAfold web server (http://rna.tbi.univie.ac.at/cgi-bin/RNAWebSuite/RNAfold.cgi, accessed on 3 July 2024) and discarded any crRNA whose spacer formed a stem-loop with a stem exceeding four consecutive base pairs. Structural analysis showed that the spacers of LbCas12a–crRNAs S1 and S3 formed such stem-loops and had lower GC contents than that of Ex4, although all three crRNAs retained intact repeat regions. Despite these structural features, LbCas12a–crRNAs S1 and S3 exhibited robust nuclease activity, and their cleavage efficiency surpassed that of Ex4, whose spacer lacked a predicted secondary structure. Specifically, RNP complexes containing S1 or S3 achieved complete template digestion in vitro, whereas RNP-Ex4 left residual uncleaved DNA (Figure 3). Based on these results, crRNA S3 was selected for subsequent HDR experiments using ssODN donors.

### 3.2. Effects of PAM Mutation and Distal Homology Arm Placement on HDR Efficiency

To assess how ssODN architecture influences CRISPR-Cas12a-mediated editing, we designed three donor variants: DHA (distal left homology arm), LP (wild-type PAM), and LT (engineered PAM mutation) (Figure 2). All editing experiments were performed using preassembled LbCas12a–crRNA–S3 RNP complexes.

**Figure 4:**
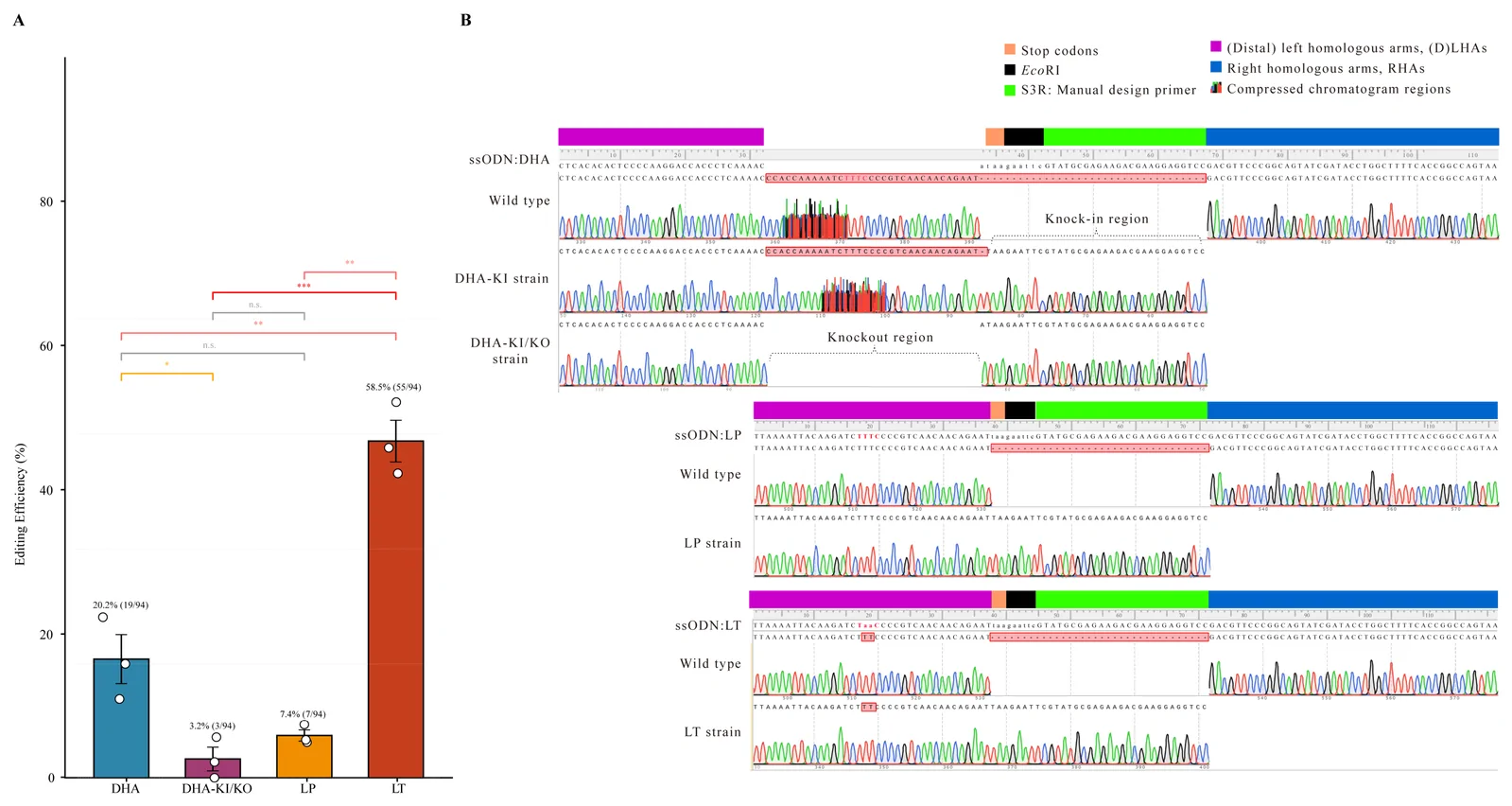
Analysis of editing efficiency and types mediated by ssODNs DHA, LP, and LT. (**A**) PCR-based assessment of gene knock-in/knock-out efficiency using primers S3F/S3R. Exact *p*-values are provided in Table S1. Significance levels: n.s., not significant; * *p* < 0.05; ** *p* < 0.01; *** *p* < 0.001. (**B**) Sanger sequencing validation of the editing events.

Quantitative analysis revealed marked differences in editing efficiency among the designs. The LT ssODN, which carried an artificial PAM mutation, yielded the highest efficiency of 58.5% (55/94 colonies; 17/32, 19/33, and 19/29 across three replicates). The DHA design, with its left homology arm positioned distally (174–205 bp upstream of the Cas12a cleavage site), showed an intermediate efficiency of 20.2% (19/94; 6/30, 8/28, 5/36). In contrast, the LP design, bearing the wild-type PAM, exhibited minimal editing activity at 7.4% (7/94; 3/32, 2/32, 2/30) (Figure 4A). These results highlight the critical role of strategic ssODN design, particularly PAM modification, in enhancing Cas12a editing outcomes.

To further characterize the editing products, we cloned and sequenced the PCR amplicons. Among the DHA-edited colonies, all harbored knock-in (KI) mutations, of which a small subset (3.2%, 3/94; 0/30, 2/28, and 1/36 across three replicates) also contained knockout (KO) mutations. Notably, all successfully modified colonies generated using the LP and LT architectures carried exclusively KI mutations (Figure 4A,B). Nevertheless, Sanger sequencing revealed that the PAM-disrupting point mutation originating from the LT ssODN template was absent in the corresponding edited transformants. The mechanistic basis underlying this observation remains to be elucidated in future experimental investigations.

### 3.3. The Influence of the RPSs on HDR Efficiency

To determine whether the recombinase *RAD51* is present in *F. filiformis*, we performed gene annotation coupled with qPCR analysis. Bioinformatic analysis of the *F. filiformis* genome identified a putative *RAD51* homolog (gene ID: EVM0007736) encoding a 340-amino acid protein. Domain annotation revealed that this protein contains a conserved RecA-like ATP-binding domain (PROSITE profile, match score = 50.60; SMART, E-value < 1 × 10^−57^), which is characteristic of the *RecA*/*RAD51* family of DNA repair and recombination proteins (NCBIfam). Multiple database entries (including FunFam, PANTHER, PIRSF, ProSiteProfiles, and Pfam) consistently annotated this sequence as a DNA repair protein RAD51 homolog (*FfRAD51*), with strong support across all major domain prediction platforms (E-values ≤ 1 × 10^−33^) (Figure S2A).

Subcellular localization prediction indicated a nuclear localization for *FfRAD51*, consistent with its expected role in nuclear DNA repair processes (Figure S2B). Furthermore, qRT-PCR analysis confirmed that *FfRAD51* is detectably expressed under standard growth conditions. The relative expression level was detectable and comparable to housekeeping genes, indicating active transcription of the *RAD51* locus (Figure S2C).

Collectively, these results demonstrate that *F. filiformis* possesses a canonical, nuclear-localized RAD51 recombinase that is both genomically encoded and transcriptionally active. This provides a molecular foundation for HDR in this species and an opportunity to incorporate RAD51-preferred motifs into ssODN donors to potentially enhance HDR efficiency in subsequent genome editing experiments.

Building on this, we analyzed three distinct constructs to assess whether RPS incorporation into ssODN donors influences editing efficiency in CRISPR-LbCas12a-mediated systems. Only the RDHA construct produced both KI and KO events, achieving a total editing efficiency of 44.7% (42/94; 13/32, 15/33, 14/29), with KO events accounting for 10.6% (10/94; 2/32, 5/33, 3/29). In contrast, the RLP and RLT constructs yielded exclusively KI mutations, with efficiencies of 28.7% (27/94; 10/32, 8/33, 9/29) and 22.3% (21/94; 3/32, 11/32, 7/30), respectively.

**Figure 5:**
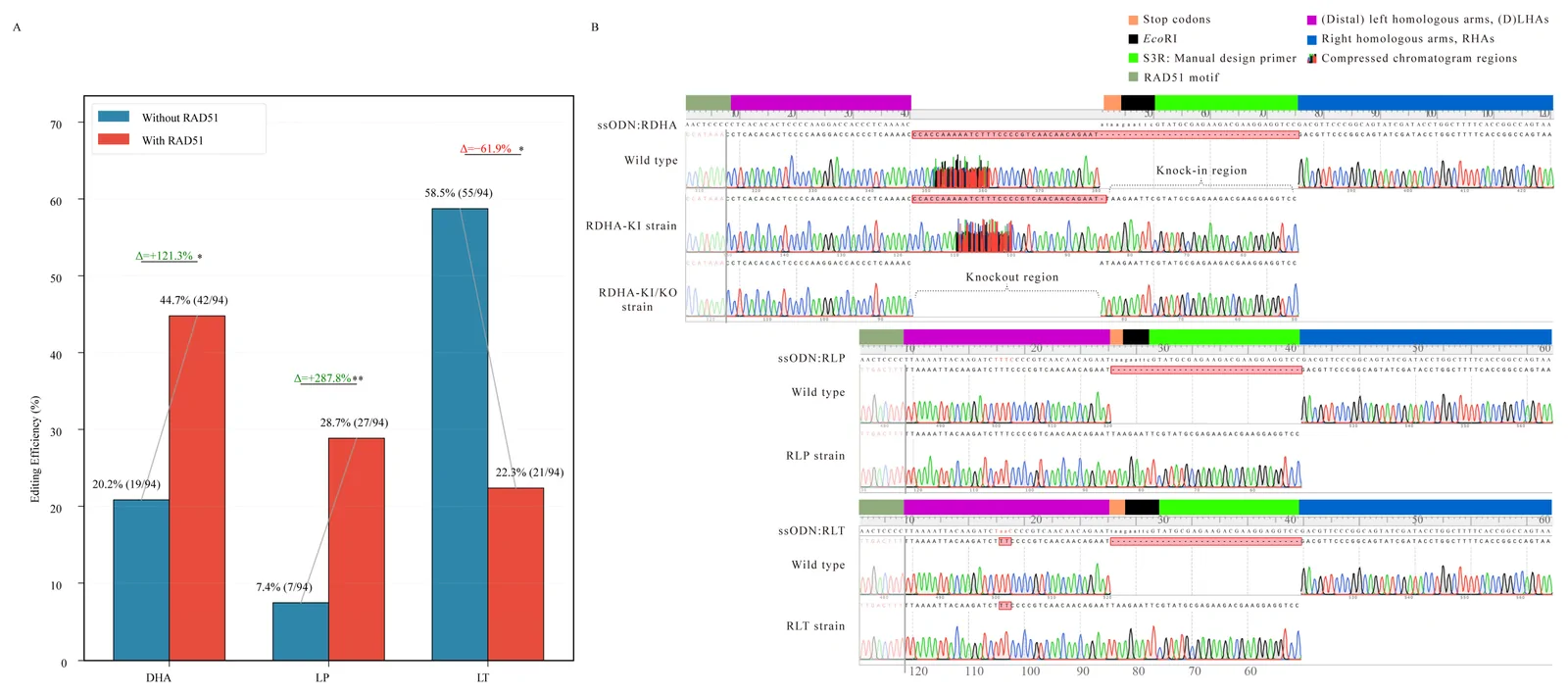
Effect of RAD51motif on ssODN-mediated gene editing efficiency in *F. filiformis*. (**A**) Comparison of gene editing efficiency among three ssODN templates (RDHA, RLP, RLT) with or without RAD51. Exact *p*-values are provided in Table S1. Significance levels: * *p* < 0.05; ** *p* < 0.01. (**B**) Sanger sequencing verification of precise knock-in and knock-out events.

Inclusion of RPSs had a highly sequence-dependent effect. Compared with the corresponding unmodified designs (Figure 4A), this inclusion increased total editing efficiency by 121.3% for DHA (from 20.2% to 44.7%) and by 287.8% for LP (from 7.4% to 28.7%), but decreased it by 61.9% for LT (from 58.5% to 22.3%). These results indicate that the effects of RPS addition are context-specific, and its application must be tailored to the target sequence rather than applied universally (Figure 5).

### 3.4. tCTS Motifs Reduce HDR Efficiency

To evaluate the influence of appended tCTS motifs on CRISPR-Cas12a editing efficiency, we designed two ssODN donors: the unmodified RLP and a modified version, RLPcts. The RLPcts construct carries two RAD51-preferred sequences and a truncated Cas12a target sequence (tCTS) at its 5′ end, together with an edge sequence and an inverted tCTS at its 3′ end (Figure 2). Both donors were tested under identical conditions using preassembled LbCas12a–crRNA-S3 RNP complexes.

**Figure 6:**
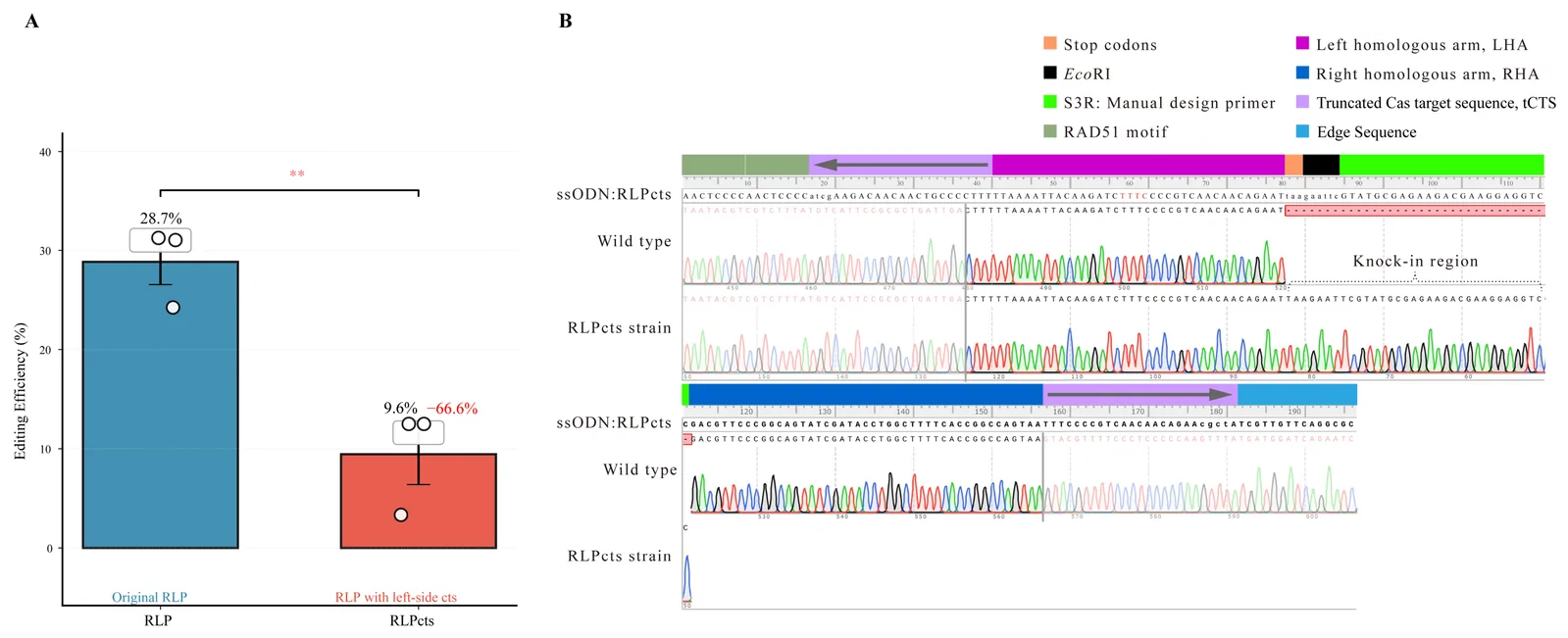
Effect of truncated LbCas12a target sequence (tCTS) on ssODN-RLP-mediated gene editing efficiency. (**A**) Comparison of editing rates between the original ssODN RLP and the ssODN RLP containing a truncated LbCas12a target sequence. Exact *p*-values are provided in Table S1. Significance levels: ** *p* < 0.01. (**B**) Sanger sequencing validation of the editing events.

Quantitative comparison revealed that introducing tCTS motifs substantially impaired editing efficiency (Figure 6). The unmodified RLP donor yielded an editing efficiency of 28.7% (27/94; 10/32, 8/33, 9/29), whereas the RLPcts construct exhibited a marked reduction, reaching only 9.6% (9/94; 4/32, 4/32, 1/30). This corresponds to a relative decrease of 66.6% compared with the control.

### 3.5. RS-1 Broadly Inhibits Cas12a-Mediated HDR

**Figure 7:**
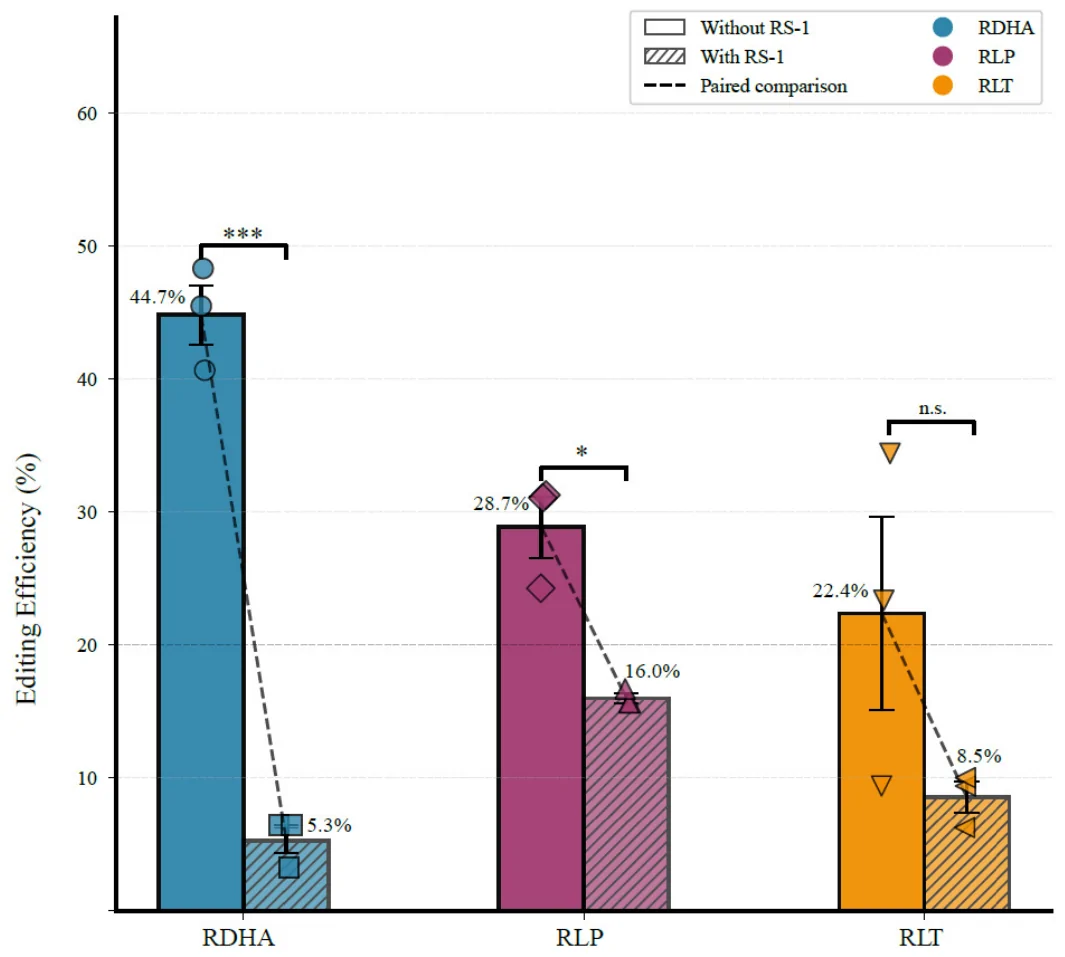
RS-1 modulates HDR efficiency in LbCas12a-mediated gene editing with different ssODN donors. Exact *p*-values are provided in Table S1. Significance levels: n.s., not significant; * *p* < 0.05; *** *p* < 0.001.

To systematically evaluate the impact of RS-1 on CRISPR-Cas12a editing efficiency, we tested three ssODN architectures (RDHA, RLP, and RLT) in the presence or absence of RS-1 supplementation (Figure 7). All experiments were performed in triplicate using preassembled LbCas12a–crRNA-S3 RNP complexes under identical conditions.

Quantitative analysis revealed that RS-1 substantially impaired editing efficiency across all constructs. In the absence of RS-1, the RDHA, RLP, and RLT donors yielded efficiencies of 44.7%, 28.7%, and 22.3%, respectively (see Section 3.3). Upon RS-1 addition, these efficiencies dropped markedly: to 5.3% for RDHA (5/94; 2/32, 2/32, 1/30), to 16.0% for RLP (15/94; 5/32, 5/32, 5/30), and to 8.5% for RLT (8/94; 3/32, 2/32, 3/30), corresponding to relative reductions of approximately 88%, 44%, and 62%, respectively. The consistent suppression observed across all three ssODN architectures at the single tested concentration, suggesting that RS-1 may act as an inhibitor (rather than an enhancer) of Cas12a-mediated HDR under these experimental conditions.

### 3.6. ΔG Does Not Significantly Affect Editing Efficiency

**Figure 8:**
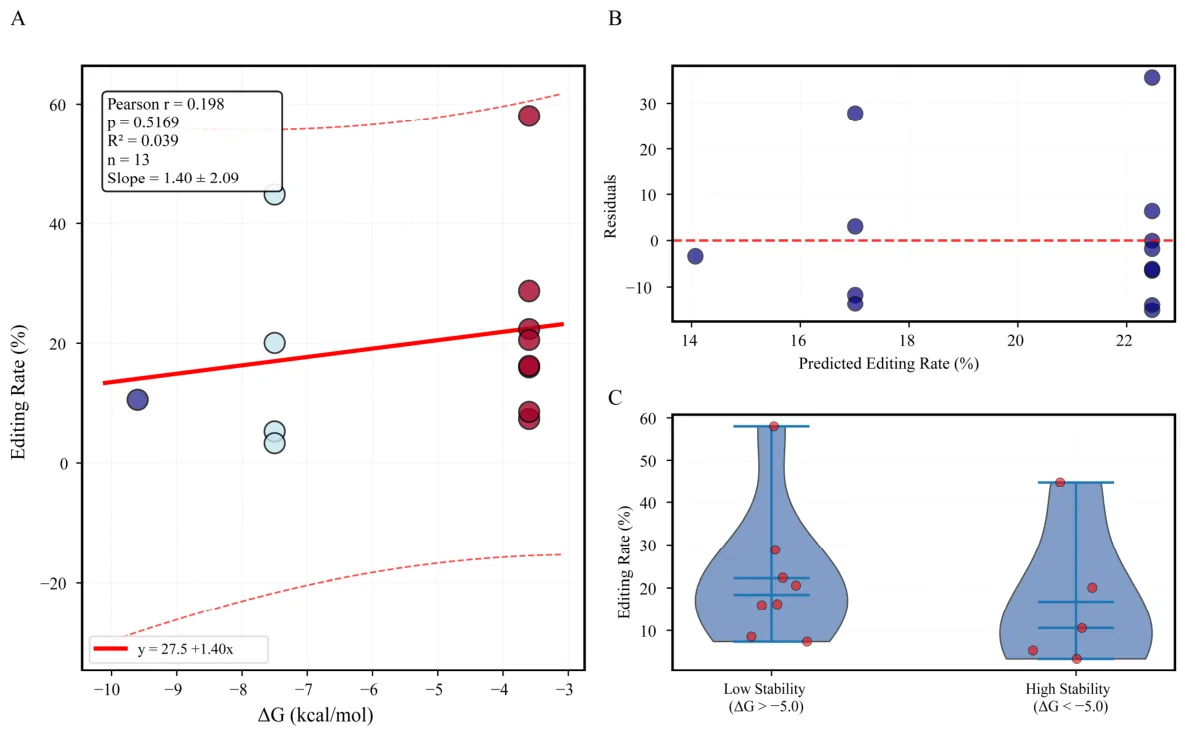
Association of ssODN ΔG with gene editing efficiency. (**A**) Scatter plot of editing efficiency versus ΔG with linear regression line (solid red) and 95% prediction intervals (dashed red). Statistical insets report Pearson correlation coefficient (r = 0.198, *p* = 0.517), coefficient of determination (R^2^ = 0.039), and sample size (n = 13). (**B**) Residual plot from linear regression analysis showing deviation from normality (Shapiro–Wilk *p* = 0.016). (**C**) Violin plots comparing editing efficiencies between low-stability (ΔG > −5.0 kcal/mol, n = 8) and high-stability (ΔG < −5.0 kcal/mol, n = 5) sequences, with individual data points overlaid. Statistical summary table provides comprehensive metrics for all analyses performed.

The statistical analyses presented in Figure 8 revealed no significant relationship between thermodynamic stability (ΔG) and editing efficiency. Both correlation and group comparisons indicated that, across the range of −9.6 to −3.6 kcal/mol, ΔG did not predict editing outcomes under the tested conditions.

Linear regression showed a weak positive trend between ΔG and editing efficiency, but this association was not statistically significant. The Pearson correlation coefficient was 0.198 (*p* = 0.517), indicating negligible linear dependence (Figure 8A). The residual plot confirmed the poor fit of the linear model. The non-random distribution of residuals and a significant Shapiro–Wilk test (*p* = 0.016) indicated violation of the normality assumption, thereby compromising the validity of parametric inferences from linear regression (Figure 8B).

When sequences were grouped by ΔG (>−5.0 vs. <−5.0 kcal/mol), editing efficiency did not differ significantly between the two groups (*p* = 0.573). The mean difference of 5.4 percentage points (22.23% vs. 16.80%) was not statistically significant, and the small effect size (Cohen’s d = 0.33) fell below the threshold for practical relevance (Figure 8C). However, given the small sample size in this study, the analytical results are presented as preliminary findings, and further conclusions await validation with additional experimental datasets.

## 4. Discussion

Unlike the diploid cellular architecture of animals and plants, the vegetative mycelia of *F. filiformis* predominantly exist in a dikaryotic heterokaryotic state. This fundamental cytological difference significantly hinders the direct transfer of well-established genome-editing workflows developed for plant and animal systems to this fungus. Although transformative tools such as CRISPR-Cas9 have achieved landmark advances in human therapeutics and crop germplasm innovation, precision genome engineering of edible mushrooms, such as *F. filiformis*, still lags far behind that of other eukaryotes.

Published studies on gene editing in *F. filiformis* remain scarce. Existing CRISPR-Cas9 platforms are limited to generating simple *pyrG* deletions or small insertions and deletions (indels); high-fidelity genome modification mediated by HDR has not been reliably achieved. CRISPR-Cas12a, which generates staggered DNA DSBs and is theoretically advantageous for promoting HDR, had not been tested in this edible fungus prior to the present work. More importantly, critical design parameters governing ssODN donors, including PAM-site disruption, the distance between homology arms and cleavage sites, RPSs, tCTSs, and the HDR-enhancing small molecule RS-1, have not been systematically characterized in any dikaryotic basidiomycete. This knowledge gap severely impedes the development and widespread application of precise genome editing across edible mushroom species.

Here, we established an LbCas12a RNP-driven HDR editing system in *F. filiformis* protoplasts and systematically dissected how diverse ssODN design parameters modulate editing efficiency. After multi-dimensional optimization, we achieved precise targeted gene edits, attaining a maximum HDR efficiency of 58.5% with PAM-mutated LT-type ssODN donors. These results verify the robust editing performance of LbCas12a in *F. filiformis* cells and provide a solid technical framework for high-precision genome engineering of this commercially important fungus. Notably, several core observations from our editing assays deviate sharply from findings reported in mammalian and plant models. These discrepancies reflect the unique genetic and repair characteristics of dikaryotic fungi and offer a valuable reference for optimizing genome-editing strategies in other heterokaryotic edible mushrooms.

First, we identified a constitutively nuclear-expressed *RAD51* gene in *F. filiformis*, implying a conserved role in genome stability maintenance. However, fusing RPS motifs to the 5′ terminus of ssODN donors exerted bidirectional, context-dependent effects on HDR efficiency. This finding supports evidence that ssDNA-dependent homologous repair can proceed independently of RAD51 activity [23] and demonstrates that simply appending RPSs is not a universal strategy for boosting HDR efficiency across eukaryotic systems [21].

Second, consistent with findings in most model systems, introducing PAM-disrupting mutations into ssODN templates dramatically elevated HDR efficiency from 7.4% to 58.5%, validating that abolishing repeated Cas-mediated cleavage of repaired loci is an effective optimization approach [24,25]. Unexpectedly, Sanger sequencing confirmed that engineered PAM mutations were not found in edited progeny, in stark contrast to the stable heritability of such mutations documented in animal and plant studies [26,27,28]. We hypothesize that this discrepancy may stem from the heterokaryotic nature of *F. filiformis*, wherein each nucleus within a cell harbors only one set of chromosomes. During DSB-triggered HDR, ssDNA acts as a linker scaffold to recruit genomic fragments flanking the break sites. In the course of genome repair, the endogenous wild-type genomic sequence serves as the primary repair template. Only if the aligned region between ssDNA and the genomic fragment lacks the native genomic sequence will the ssDNA template sequence be utilized for repair. Further mechanistic validation is required to substantiate the observations and hypotheses presented herein. Meanwhile, these findings underscore the necessity of conducting additional experiments under heterokaryotic conditions to expand our understanding of DNA DSB repair modes.

Third, tCTS addition to ssODN donors, an approach reported to strengthen HDR efficiency in multiple plant and mammalian systems [29,30], significantly inhibited editing efficiency in our enokitake platform. We propose three potential underlying causes: (1) tCTS sequences may exert cytotoxicity on fungal protoplasts [31]; (2) additional tCTS fragments may promote complex secondary structures within ssODNs, impeding pairing between homology arms and genomic templates; and (3) tCTS-mediated enhancement may be more suitable for double-stranded DNA donors that are recognized and transported to DSBs by Cas RNPs after pre-incubation. These results establish a new decision-making framework for ssODN design in edible fungi and caution researchers to thoroughly evaluate donor type, Cas pre-assembly, nuclear localization signal configuration, and cleavage susceptibility before incorporating tCTS elements.

Fourth, our work expands the acceptable distance between homology arms and DSB sites for ssODN-mediated HDR. While previous studies recommended positioning homology arms within 10 nt of breakpoints, with an upper limit of approximately 100 nt [32,33], we retained 20.2% HDR efficiency when the left homology arm was spaced 174 nt from the cleavage site. This finding broadens the design flexibility of homology arm placement in edible fungi.

Fifth, contrary to its well-documented role as a RAD51 filament stabilizer [34], RS-1 failed to stimulate HDR in *F. filiformis*. Instead, 12 μmol/L RS-1 generated a pronounced inhibitory effect across all treatment groups, in direct contrast to its HDR-promoting function in porcine and other animal models [35,36] and differing from the mild, non-significant improvements observed in certain other eukaryotic systems [37,38]. We infer that species-specific regulatory differences in RAD51 or suboptimal RS-1 concentrations for fungal cells account for this suppression, reinforcing that editing schemes optimized for animal cells cannot be directly transferred to dikaryotic basidiomycetes without re-optimization.

Beyond these core parameters, we implemented several auxiliary optimization measures: phosphorothioate modification at the two or three terminal nucleotides of ssODNs and crRNAs to enhance intracellular stability [39], and stringent crRNA screening to ensure efficient DSB induction while maintaining full accessibility of homology arms. The combination of these multi-layered strategies jointly underpinned the high precision and efficiency of our Cas12a editing system. Collectively, all divergent outcomes between this fungal study and plant/mammalian research imply that heterokaryotic edible fungi possess unique homologous repair regulatory networks. Editing strategies optimized for higher eukaryotes cannot be mechanically copied; species-specific donor design and editing optimization standards must be established for dikaryotic fungi.

This study systematically dissected how ssODN donor properties influence LbCas12a-mediated HDR efficiency in *F. filiformis*. We found that introducing PAM-disrupting mutations into ssODN templates robustly elevated HDR efficiency; however, in stark contrast to the stable heritability of such mutations documented in previous animal and plant studies, these mutations were lost in edited progeny. We also found that a homology arm could remain functional at a distance of 174 bp from the DSB, substantially extending the known spatial tolerance. The effect of RPSs proved highly context-dependent, enhancing HDR in certain donor designs while paradoxically suppressing it in others, whereas tCTS motifs and RS-1 consistently acted as negative regulators. Furthermore, no simple linear correlation was observed between ssODN ΔG and editing outcomes. These results establish refined, context-aware design principles for ssODN donors and provide a rational framework for donor optimization. This work lays a critical foundation for precise genome editing in *F. filiformis* and offers tentative, transferable insights for genetic manipulation of other heterokaryotic fungi.

That said, the present study has several inherent limitations. All experimental results were obtained using strain 22Y and only tested a single genomic target locus. Therefore, further independent experimental validation is required before these ssODN design principles can be generalized to other genomic sites or other heterokaryotic basidiomycete species. Despite these constraints, our findings deliver actionable empirical rules to guide ssODN-mediated genome-editing attempts in this edible mushroom.

## Figures and Tables

**Table 1:** Sequences and design parameters of genes and ssODN donors used in CRISPR-LbCas12a-mediated gene editing.

Genes/ssODNs	Primer or ssODN Sequences (5′-3′)	Product Length (bp)
*S3F/S3R* ^a^	ACTCTTCTTCTTCGCCGCCAGT/GGACCTCCTTCGTCTTCTCGCATA	166/353
*Rpb2*	GGACGCAGAAGAGGAGGAGACT/CTTGAGCCGAGCAGCAGGAT	130
*CytB*	GCTGCTTTAGTTGTGGCACATT/AGGTGTAACCATTGGATCTGCT	228
*RAD51*	GGTATTGCCGTTGTCGTCAC/CTTCTTCTCATTGCCTGCGTAA	81
DHA ^b^	CTCACACACTCCCCAAGGACCACCCTCAAAAC**ataagaattc**GTATGCGAGAAGACGAAGGAGGTCCGACGTTCCCGGCAGTATCGATACCTGGCTTTTCACCGGCCAGTAA	112
LP ^b^	TTAAAATTACAAGATCTTTCCCCGTCAACAACAGAAT**taagaattc**GTATGCGAGAAGACGAAGGAGGTCCGACGTTCCCGGCAGTATCGATACCTGGCTTTTCACCGGCCAGTAA	116
LT ^b^	TTAAAATTACAAGATC**Taa**CCCCGTCAACAACAGAAT**taagaattc**GTATGCGAGAAGACGAAGGAGGTCCGACGTTCCCGGCAGTATCGATACCTGGCTTTTCACCGGCCAGTAA	116
RDHA ^b^	**AACTCCCCC**TCACACACTCCCCAAGGACCACCCTCAAAAC**ataagaattc**GTATGCGAGAAGACGAAGGAGGTCCGACGTTCCCGGCAGTATCGATACCTGGCTTTTCACCGGCCAGTAA	120
RLP ^b^	**AACTCCCC**TTAAAATTACAAGATCTTTCCCCGTCAACAACAGAAT**taagaattc**GTATGCGAGAAGACGAAGGAGGTCCGACGTTCCCGGCAGTATCGATACCTGGCTTTTCACCGGCCAGTAA	124
RLT ^b^	**AACTCCCC**TTAAAATTACAAGATC**Taa**CCCCGTCAACAACAGAATt**aagaattc**GTATGCGAGAAGACGAAGGAGGTCCGACGTTCCCGGCAGTATCGATACCTGGCTTTTCACCGGCCAGTAA	124
RLPcts ^b^	**AACTCCCCAACTCCCC**ATCGAAGACAACAACTGCCCCTTTTTAAAATTACAAGATCTTTCCCCGTCAACAACAGAAT**taagaattc**GTATGCGAGAAGACGAAGGAGGTCCGACGTTCCCGGCAGTATCGATACCTGGCTTTTCACCGGCCAGTAA**TTTCCCCGTCAACAACAGAACGCTA**TCGTTGTTCAGGCGC	196

^a^ The ssODN- mediated knockout and knock-in strains yielded PCR products of 166 bp and 353 bp, respectively. ^b^ All ssODNs have phosphorothioate modifications on the two terminal nucleotides at both the 5′ and 3′ ends. Bold formatting highlights key functional motifs: stop-codon (taa); *Eco*RI restriction site (gaattc); mutated PAM (Taa); RAD51-preferred motif (AACTCCCC); and cts fragment (ATCGAAGACAACAACTGCCCCTTT). Abbreviations: DHA, distal left homology arm; LP, wild-type PAM; LT, engineered PAM mutation; CTS, truncated Cas target sequence; RDHA, RAD51-DHA; RLP, RAD51-LP; RLT, RAD51-LT. RLPcts consists of RLP, two tCTS fragments, and a random 16-base sequence (5′-ATCGTTGTTCAGGCGC-3′).

## Data Availability

The original contributions presented in this study are included in the article/Supplementary Materials. Further inquiries can be directed to the corresponding authors.

## Supplementary Materials

The following supporting information can be downloaded at: https://www.mdpi.com/article/10.3390/jof12090705/s1, Figure S1: Structure and associated sites of the color-trait-related tyrosinase gene (EVM0009893). Figure S2: Bioinformatic analysis of the RAD51 protein from *F. filiformis*. (A) Conserved domain annotation and architecture. (B) Subcellular localization prediction. (C) Relative RAD51 expression level analysis. Table S1: Exact *p*-values and statistical parameters for all pairwise comparisons shown in Figure 4, Figure 5, Figure 6 and Figure 7.
