# Supplementary material for: Systematic Profiling of ssODN Determinants Establishes Optimized LbCas12a-Mediated HDR for Precise Genome Editing in Flammulina filiformis
Source: J Fungi (Basel). 2026 Sep 21;12(9):705. doi: 10.3390/jof12090705 (PMC13608477; doi:10.3390/jof12090705)
Supplement: Supplementary file 1 [file jof-12-00705-s001.zip › Figure S1. Structure and associated sites of the color-trait-related tyrosinase gene (EVM0009893).pdf]

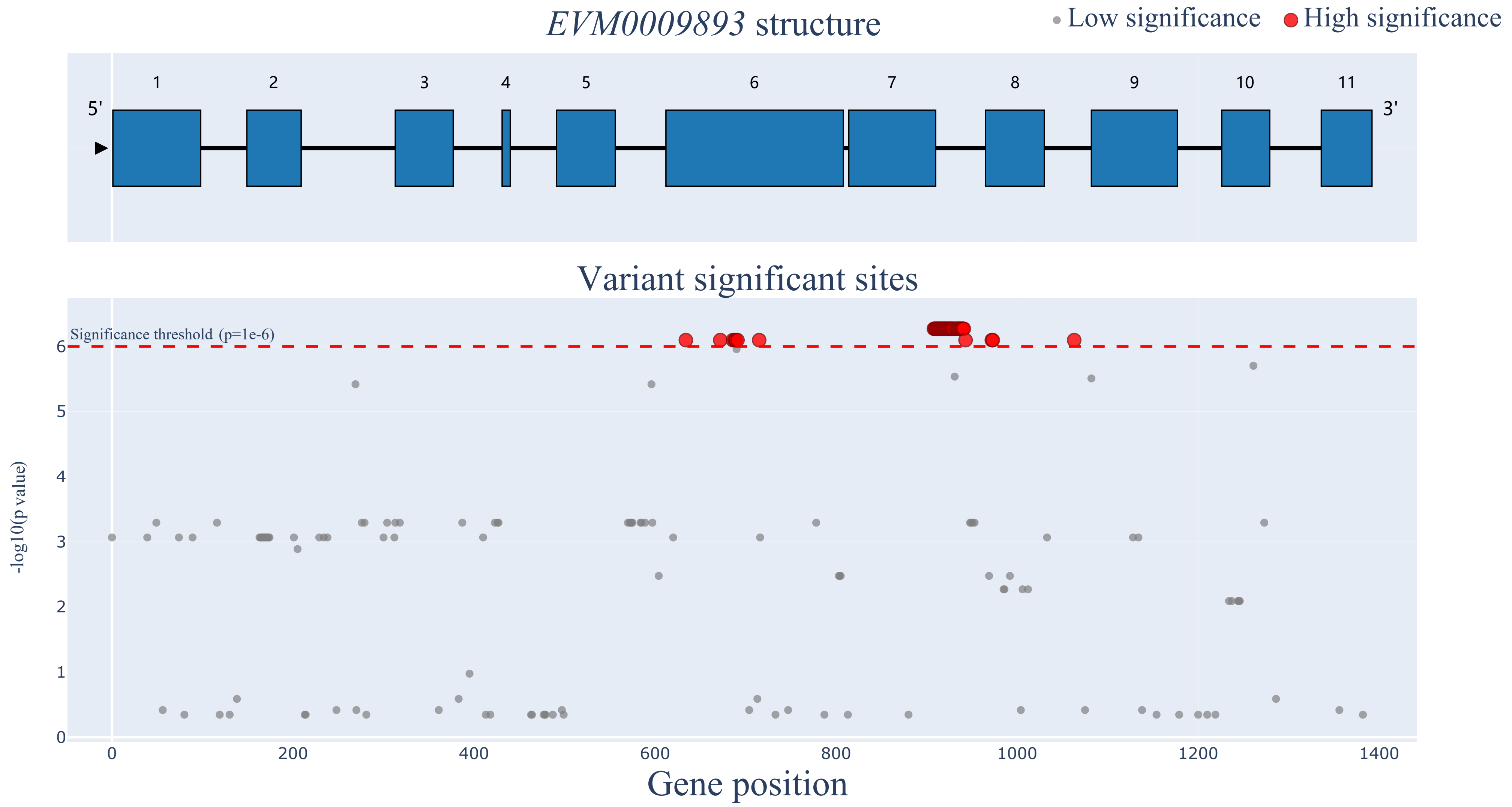

EVM0009893 structure
Low significance
High significance
1
2
3
4
5
6
7
8
9
10
11
5'
3'
Variant significant sites
Significance threshold (p=1e-6)
-log10(p value)
0
1
2
3
4
5
6
0
200
400
600
800
1000
1200
1400
Gene position
