# Supplementary material for: Systematic Profiling of ssODN Determinants Establishes Optimized LbCas12a-Mediated HDR for Precise Genome Editing in Flammulina filiformis
Source: J Fungi (Basel). 2026 Sep 21;12(9):705. doi: 10.3390/jof12090705 (PMC13608477; doi:10.3390/jof12090705)
Supplement: Supplementary file 1 [file jof-12-00705-s001.zip › Table S1.pdf]

Table S1. Exact p-values and statistical parameters for all pairwise comparisons shown in Figures 4-7.

Comparison	Mean A (%)	Mean B (%)	Abs. Δ (%)	Rel. Δ (%)	t-statistic	p-value	Sig.	Figures
DHA vs. DHA-KO	20.8	3.3	−17.5	—	3.7	0.0363	*	Figure 4
DHA vs. LP	20.8	7.4	−13.4	—	3.06	0.0812	n.s.	Figure 4
DHA vs. LT	20.8	58.7	37.9	—	6.78	0.0027	**	Figure 4
DHA-KO vs. LP	3.3	7.4	4.1	—	—	>0.10	n.s.	Figure 4
DHA-KO vs. LT	3.3	58.7	55.4	—	13.27	0.0007	***	Figure 4
DHA vs. RDHA (−RPS vs. +RPS)	20.1	44.8	24.7	123.30%	—	0.039	*	Figure 5
LP vs. RLP (−RPS vs. +RPS)	7.4	28.8	21.4	289.60%	—	0.008	**	Figure 5
LT vs. RLT (−RPS vs. +RPS)	58	22.4	−35.6	−61.4%	—	0.03	*	Figure 5
RLP vs. RLPcts	28.8	9.4	−19.4	−67.3%	5.071	0.0086	**	Figure 6
RDHA −RS-1 vs. +RS-1	44.8	5.3	−39.5	−88.2%	—	0.0009	***	Figure 7
RLP −RS-1 vs. +RS-1	28.8	16	−12.9	−44.6%	—	0.0282	*	Figure 7
RLT −RS-1 vs. +RS-1	22.4	8.5	−13.8	−61.8%	—	0.1936	n.s.	Figure 7
